# Blended learning across universities in a South–North–South collaboration: a case study

**DOI:** 10.1186/s12961-016-0136-x

**Published:** 2016-09-02

**Authors:** Myroslava Protsiv, Senia Rosales-Klintz, Freddie Bwanga, Merrick Zwarenstein, Salla Atkins

**Affiliations:** 1 Department of Public Health Sciences (Global Health/IHCAR), Karolinska Institutet, Tomtebodavägen 18A, 171 77 Stockholm, Sweden; 2 College of Health Sciences, Makerere University, Kampala, Uganda; 3 Department of Family Medicine, Schulich School of Medicine and Dentistry, Western University, 1151 Richmond St, N6A 3K7 London, Ontario Canada; 4 Faculty of health sciences, Stellenbosch University, Francie Van Zijl Dr, Tygerberg Hospital, Cape Town, 7505 South Africa

**Keywords:** Blended learning, e-learning, Higher education, Doctoral training, Research capacity building, Health services, Health systems

## Abstract

**Background:**

Increased health research capacity is needed in low- and middle-income countries to respond to local health challenges. Technology-aided teaching approaches, such as blended learning (BL), can stimulate international education collaborations and connect skilled scientists who can jointly contribute to the efforts to address local shortages of high-level research capacity. The African Regional Capacity Development for Health Systems and Services Research (ARCADE HSSR) was a European Union-funded project implemented from 2011 to 2015. The project consortium partners worked together to expand access to research training and to build the research capacity of post-graduate students. This paper presents a case study of the first course in the project, which focused on a meta-analysis of diagnostic accuracy studies and was delivered in 2013 through collaboration by universities in Uganda, Sweden and South Africa.

**Methods:**

We conducted a mixed-methods case study involving student course evaluations, participant observation, interviews with teaching faculty and student feedback collected through group discussion. Quantitative data were analysed using frequencies, and qualitative data using thematic analysis.

**Results:**

A traditional face-to-face course was adapted for BL using a mixture of online resources and materials, synchronous online interaction between students and teachers across different countries complemented by face-to-face meetings, and in-class interaction between students and tutors. Synchronous online discussions led by Makerere University were the central learning technique in the course. The learners appreciated the BL design and reported that they were highly motivated and actively engaged throughout the course. The teams implementing the course were small, with individual faculty members and staff members carrying out many extra responsibilities; yet, some necessary competencies for course design were not available.

**Conclusions:**

BL is a feasible approach to simultaneously draw globally available skills into cross-national, high-level skills training in multiple countries. This method can overcome access barriers to research methods courses and can offer engaging formats and personalised learning experiences. BL enables teaching and learning from experts and peers across the globe with minimal disruption to students’ daily schedules. Transforming a face-to-face course into a blended course that fulfils its full potential requires concerted effort and dedicated technological and pedagogical support.

## Background

Low- and middle-income countries (LMICs) in sub-Saharan Africa (SSA) face the heaviest disease burden in the world but have weak health systems and the least resources to meet these challenges [1]. In particular, these countries have a dearth of skilled researchers who can perform high-quality, relevant research to inform health policy. Local universities should play a key role in building this research capacity and equipping young researchers with the necessary skills to contribute to their home countries’ health systems [2–5]. However, African universities struggle to respond to this need due to the novelty of the health systems and services research (HSSR) field, the lack of established curricula and the limited pool of high-skilled teaching staff to deliver training [3, 4]. The annual output of doctoral programmes focusing on HSSR at the four major medical universities in SSA is five graduates, or approximately 4% of all doctoral graduates – a rate unlikely to be sufficient to support policy changes in health systems [6].

Sandwich programmes, which typically combine coursework and meetings with supervisors at northern universities with research conducted in the student’s home country [7], can build young researchers’ capacity while encouraging them to continue working in their local health systems [8]. However, these programmes can engage only a limited number of doctoral students. For instance, the sandwich programme between Makerere University (MU) and Karolinska Institutet (KI) produces about three doctoral graduates per year in all disciplines [8]. Further, the travel requirement might discourage some students from participating [9]. New approaches are needed to achieve the scale and pace of building research capacity necessary to address health challenges in SSA [10].

Technology-aided learning can offer a virtual alternative to the physical travel needed for sandwich courses. Technology-aided education can increase access [11] and flexibility in the time or place where learning occurs [12] and reduce the environmental impact due to less travel [13]. However, pure online learning has some disadvantages, such as technological difficulties, alienation and the absence of a sense of community [14, 15], which can decrease learners’ motivation and increase dropout rates [16]. Blended learning (BL), broadly defined as an approach that integrates components of face-to-face and online learning [17], has the potential to counter these disadvantages.

BL has been used successfully in continuing medical education and public health professional training [18–21], but not in the HSSR disciplines [22]. The African Regional Capacity Development for Health Systems and Services Research (ARCADE HSSR) consortium decided to experiment with this method to build research capacity through engaging international academic collaborations and developing blended courses on HSSR methods. BL is a relatively new and dynamically developing research area as the approach itself is evolving [23]. Recent evidence suggests that BL produces equal or better outcomes to traditional teaching methods in public health and clinical disciplines [24–28] and that students prefer BL [29–31]. BL in health research training is under-studied, although studies on the application of BL in masters’ level studies are increasing [28–30, 32, 33]. Fewer doctoral training studies have been reported, and the existing ones in nursing disciplines employ a pure online-learning approach [34–36]. Further, there are only a few studies on doctoral-level courses on health research using BL in the grey literature [37–39], pointing to the need for more evidence of the applicability of this approach to high-level research training.

To inform future implementations of BL in ARCADE, the aim of this case study [40] was to explore the preparation and delivery of a doctoral course, the Meta-analysis of Diagnostic Accuracy Studies (MADAS). It was the first course offered by the ARCADE HSSR consortium and was delivered concurrently across three universities in low-, middle- and high-income countries (Uganda, South Africa and Sweden). This paper presents an evaluation of the course implementation and discusses the potential of BL for research capacity building.

### Course description

The MADAS course was offered in a BL format by MU from Uganda in 2013, with participation by collaborators and students from Stellenbosch University (SU) in South Africa and KI in Sweden. The course was the first experience of a southern university-led course shared across three countries.

The course targeted doctoral and post-doctoral level students in the health sciences and clinical medicine, and its aim was to introduce the concept of diagnostic accuracy studies. The course used selected examples of diagnostic tests to develop students’ skills in conducting a meta-analytic study by practicing literature searches, managing data, performing a meta-analysis using open-source software (MetaDisc^®^) [41], and interpreting the findings.

Initially, the course was conducted at MU and repeated at KI as a traditional, 1-week, classroom course taught face to face. On these occasions, it became apparent that many more students were interested in attending the course but could not do so at that time, so plans were made to offer it as a blended course with more frequent runs. The MADAS course was thus converted into BL format by the course developer (FB). The course and its learning objectives are described in Table 1. The self-directed portion of the course materials can be accessed through the ARCADE Open Course Repository [42].

**Table 1:** MADAS course description and learning objectives

Course aim	Learning outcomes	Delivery mode	Assessment methods
The aim of the course was to train doctoral students to conduct a meta-analysis of diagnostic accuracy studies (DAS), from study design to manuscript preparation	At the end of the course, the student will: 1. Understand the importance, meaning and concepts of DAS and be able to discuss the limitations, biases and challenges faced in meta-analyses 2. Develop a protocol for a meta-analysis study on DAS 3. Conduct a comprehensive search of DAS on a selected test 4. Manage data: define the variables, collect data, perform a meta-analysis of data using applicable software, and interpret the findings 5. Prepare a manuscript on a meta-analysis study for publication	Remote delivery via: 1. Synchronous tools: web conferencing and chat sessions (50% of the course) 2. Asynchronous tools: e-mail and Moodle platform Geographically co-located through local tutors at each university	The final grade was based on students’: 1. Knowledge assessment of the main concepts in the course through a multiple-choice exam 2. Submitted draft protocol of a meta-analysis study 3. Participation in discussions

#### The team

The pedagogic team included the course leader from MU (FB), two local facilitators at KI (SRK) and SU, and informational technology support staff who assisted with course implementation at each site. In addition, the ARCADE project coordinator at KI (MP) took part in the course as an active observer to evaluate this first experience within the ARCADE HSSR collaboration.

#### Course design and preparation

**Fig. 1:**
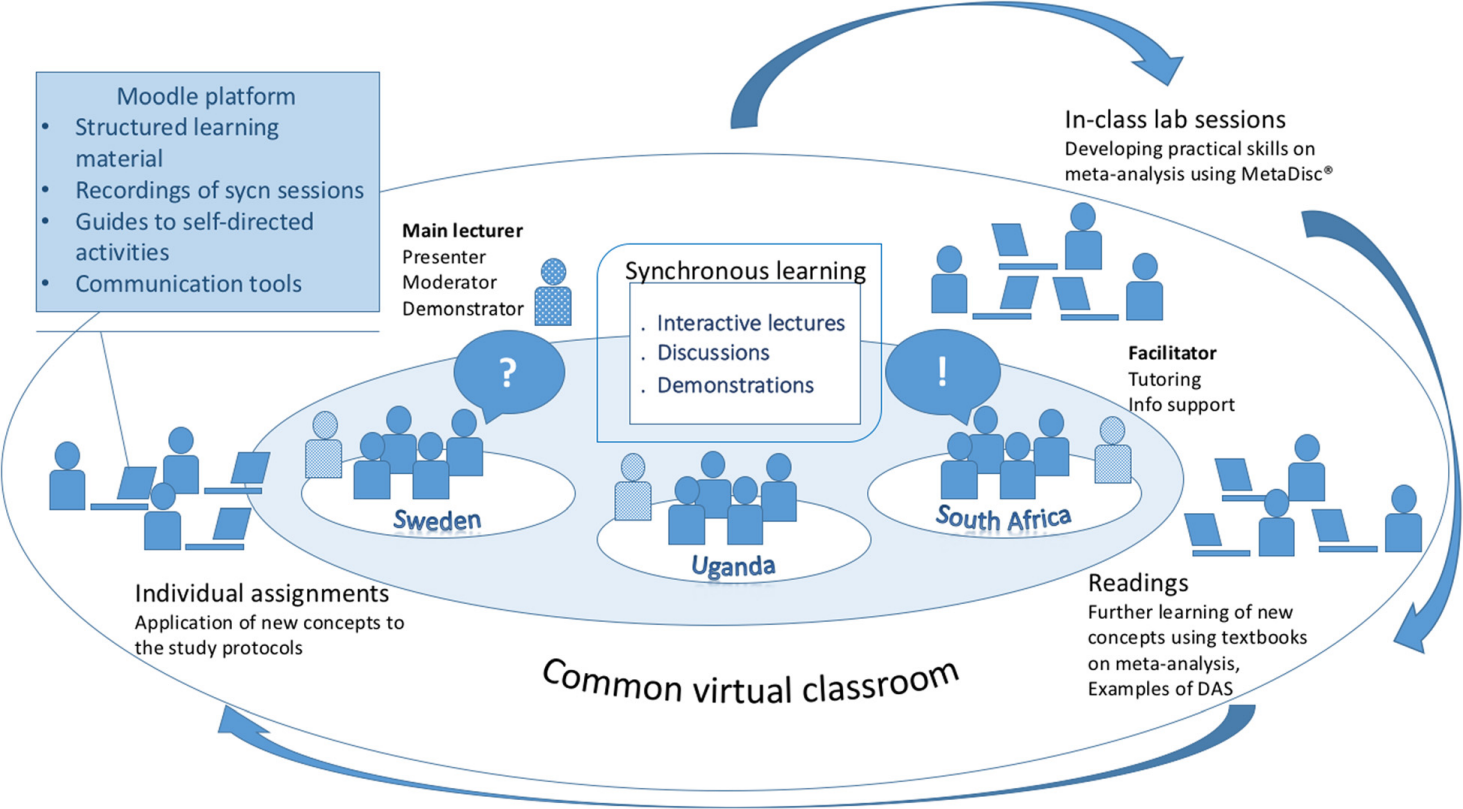
Logic model of MADAS course design

The BL course was designed as a mix of interactive, synchronous online sessions, self-directed online-learning activities, and combined online and face-to-face interactions between students and teaching staff [31, 43]. Synchronous sessions were led by the main lecturer based at MU, and in-class practical exercises and self-directed learning were supported face to face by the facilitators at the remote sites. Figure 1 depicts the logic model followed in the preparation of the course.

The existing learning materials were converted into an online format and integrated within learning activities that supported interactivity and built practical skills. During course preparation, lecture content and demonstration sessions for practical exercises were developed for online delivery. Other materials for BL included selected core readings, reference literature, syllabi and detailed schedules of synchronised, on-site and self-directed activities, and guides for individual assignments and for learning activities in each of the five course modules. The materials were organised on the Moodle platform in a module structure that directed students to complete learning activities to achieve the learning objectives.

#### Course delivery

The BL MADAS course was offered as an elective within the 2013 doctoral programmes of the participating universities: the World Health Programme at KI, the doctoral programme in Medical Sciences at MU, and the research programme at the Centre for Evidence Based Health Care and the Department of Health Sciences at SU. The course was delivered concurrently to all three universities in different countries on January 21–25, 2013. Nineteen doctoral and pre-doctoral students (i.e. waiting for formal registration in their home universities) and researchers from the three universities enrolled in the course. The group represented a diverse mix of academic backgrounds, including clinical research, microbiology, pharmacology, obstetrics, epidemiology and health systems research.

The teaching and learning activities were distributed over two calendar weeks. The first week consisted of full-time learning activities, including interactive, synchronous online sessions, practical exercises and individual self-directed learning using the course materials available online. The second week was devoted to students completing their individual assignments.

The instructor-led activities delivered during the first week were equivalent to 40 hours of full-time studies. Students from South Africa and Sweden joined synchronous online sessions every morning (8:30–13:00 Central European Time) for five consecutive days. While most students in Uganda were present for these sessions in the classroom at MU, a few students switched to online participation after learning about the possibility.

The synchronous sessions were comprised of lectures, discussions and demonstrations of meta-analysis. FB, acting as both the course leader and the main lecturer, was physically present at MU and virtually present at the other sites. He led these sessions with participation by invited guest lecturers at KI. Local facilitators were present at each site to provide face-to-face tutoring, assist students with practical tasks (e.g. literature searches, data extraction, meta-synthesis), and respond to students’ questions about the course content or modalities. Interactions between students and course faculty were complemented with real-time chatroom sessions and contact via e-mail.

Individual self-directed activities included readings and homework, which contributed to the individual assignment consisting of a study protocol that students were expected to complete and deliver after the second week of the course. Box 1 presents an overview of the content and learning activities in the course.

Students had access to online learning materials through Moodle, an open-source e-learning platform [44]. The materials included downloadable online lecture notes, PowerPoint slides, video-recorded past lectures, links to journal articles and textbooks on meta-analysis, and access to MetaDisc^®^ [41], an open-source data management and statistical analysis package for performing meta-analyses.

## Methods

Social constructivism holds that verbal interactions, reasoning and the application of knowledge to real-world contexts result in learning [45, 46]. This theory informed the course design and evaluation. We used the seven principles of effective learning based on social constructive theory [47, 48] as an analytical framework in this study (Box 2). We analysed teaching staff and students’ feedback on the course design and learning activities and used the principles as a lens through which we examined responses.

### Data collection

The data sources for the course evaluation included the routine course evaluation survey, notes from a group discussion with students, participant observation of synchronous online sessions and interviews with MADAS course teaching staff (course leaders and local facilitators) about their experiences with BL. MP conducted the group discussion and participant observation in January and February 2013.

At the end of the course, participants were contacted by email and invited to participate in an online evaluation survey. The survey included standard course-evaluation questions using a 6-point Likert scale to assess self-reported achievement of the learning objectives, knowledge gained and learning of new skills. We also included questions focusing on the experience of technology-aided learning. For the participant observation, MP attended and observed all the synchronous online sessions (n = 11) at KI and took notes about the implementation. The information collected during observation guided the questions in the group discussion. The group discussion was conducted with three students physically present on the KI campus in Sweden. The session was video-recorded, and notes were taken and expanded after watching the video. The data were complemented by teaching staff’s reflections on the course preparation and implementation during semi-structured interviews conducted in February 2014.

#### Data analysis

Results from the survey were exported into Microsoft Excel and analysed using descriptive statistical analysis. The participant observation, students’ group discussion and instructors’ interviews were transcribed verbatim, and the qualitative data was subjected to thematic analysis [49]. MP read and re-read the transcribed texts to familiarise herself with the content. MP next proposed the units of meaning, assigned them codes and organised them into categories. The categories were validated by SA, and themes were generated from the finalised categories. Triangulation was accomplished by combining several data sources and including both teaching staff and students to represent different perspectives. Detailed descriptions of the themes and illustrative examples were used to promote the trustworthiness of the qualitative analysis.

## Results

In this section, we present the results of the combined data from students’ evaluation survey and qualitative data from participant observation and students’ and teachers’ feedback. We report the findings under the following subheadings: (1) purpose of the adaptation to BL; (2) course participants; (3) students’ course evaluation; (4) experimentation with BL instructional approaches; and (5) benefits of the design and proposed changes. In addition, we use the feedback from students and teaching staff on learning activities and instructional approaches and analyse how they relate to the principles of effective teaching [47, 48]. The results are presented in Table 2 below.

**Table 2:** Demographic characteristics of survey respondents

University	Male, n (%)	Female, n (%)	Total, n (%)
Makerere University	5 (62)	3 (38)	8 (44)
Stellenbosch University	2 (50)	2 (50)	4 (22)
Karolinska Institutet^a^	2 (33)	4 (67)	6 (33)
Total	9 (50)	9 (50)	18 (100)

^a^Of the six KI students, three were enrolled in the joint KI-MU doctoral programme and joined the course from Uganda

### Purpose of BL adaptation

The MADAS course was aimed at equipping doctoral and post-doctoral students with research skills in conducting meta-analysis, from formulating the research questions and developing a protocol to synthesising the data and preparing a report. The goal of the BL adaptation was to retain the practical approach and highly interactive nature of the original face-to-face course while meeting adult learners’ needs, maximising their learning and allowing flexibility to overcome barriers such as time, cost and travel.

### Course participants

In total, 19 students participated in the course. Of these, 18 responded to the evaluation survey (Table 3). All participants were either preparing for registration as doctoral students (n = 2), already registered as doctoral students (n = 14), or were postdoctoral or senior researchers (n = 4).

**Table 3:** Implementation of effective teaching and learning principles in the MADAS course

Theoretical construct	Instructional method	Experience and lessons learned (SF, student feedback; TF, teaching staff feedback)
1. Student–faculty contact	Direct interaction with the main lecturer and guest speakers via synchronous online sessions	The experience of ‘live’ interaction with experts was exciting, but the occasional interruptions were frustrating (SF) During synchronous sessions, the instructor’s attention had to be divided between the face-to-face and remote online student groups (TF)
	Regular in-class and email contact with course leader and facilitators	Support for students during and between the synchronous sessions helped create a “safe blended learning environment” (SF)
2. Collaborative and social learning	Synchronous online discussions	Real-time discussions were a key to students’ peer-to-peer learning. The online modality of discussion somewhat limited the possibilities for spontaneous comments or questions (SF) Video of students in remote classrooms was not available at all times, and students wanted to know to whom they were speaking (SF)
	Collaborative assignments	Students wished for more opportunities for formal and informal interaction with other university sites (SF)
3. Active learning	Individual assignments Demonstrations	Individual assignments and practical exercises helped students apply new knowledge and kept students engaged (SF) Students wished to work on the research problems of their interests (SF)
4. Prompt feedback	Feedback via email or on site from on-site facilitators and the main course leader Interaction via synchronous online sessions	Local facilitators strengthened personalised feedback to students, which they valued (SF) These sessions enabled getting immediate answers and learning from peers (SF)
5. Time on task	Access to structured course materials and video-recorded lectures on the Moodle platform	The time allocated to self-directed learning was not sufficient to explore some learning materials and activities (SF); the duration of the course and time allocated for completing self-directed activities should be revised
6. High expectations	High expectations about quality of the final assignment set by individual assignments	Students found individual assignments to be ‘useful’ in their learning and were motivated to submit high-quality assignments and get individualised comments on their work (SF)
7. Respect for diverse talents and ways of learning	Combination of learning activities in the course	The combination of learning activities helped match different learning styles. Students reported different views on the most helpful learning activities in the course (readings and exercises), which might be related to their preferred learning styles (SF)
	Selected level of complexity of the learning materials	Based on students’ feedback, the level of complexity was appropriate (SF)
	Synchronous online sessions connecting people in three different contexts	The participation of students with diverse backgrounds in the discussions stimulated learning through providing interesting examples and challenging other’s ideas

### Students’ course evaluations

**Fig. 2:**
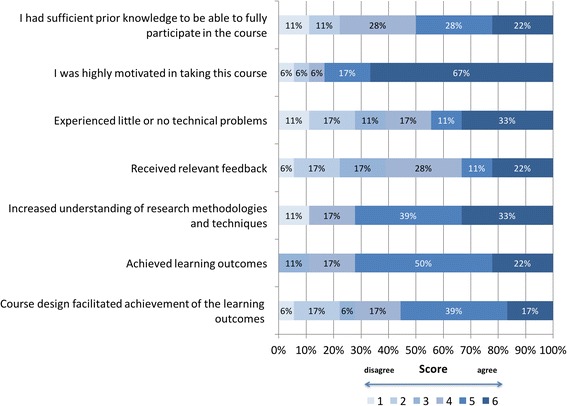
Selected items on students’ responses to the course design and achievement of the learning objectives based on 18 observations

Overall, students were satisfied with the blended course, and 94% (17 students) would recommend it to their fellow doctoral students. Most students were happy with the current mix of face-to-face and self-directed studying in the course (11 students) and experienced few or no technical problems (11 students) (Fig. 2). More detailed feedback can be found in the following subsections describing the teaching approaches and students’ responses to them.

### Experimentation with BL instructional approaches

The BL aspects of the course design supported two key goals of the course: interactivity and a focus on research skill development. The BL aspect also built flexibility of geographical location into the course design, allowing students to join the synchronous sessions from any site and offering self-directed activities that students could perform at their convenience.

Social constructivism was used as the theoretical approach to the course design. Therefore, the course was developed with the goal to provide rich interaction experiences supported by web-conferencing links among Uganda, South Africa and Sweden. The synchronous online discussion offered the possibility for a diverse group of students from three countries to exchange ideas, ask questions and discuss real-world problems, resulting in opportunities for peer-to-peer learning:

“*It* [the course] *was interactive, and I learnt through the experiences of other students from the other participating institutions.*” Evaluation survey respondent

Like the students, the facilitators felt that teaching across three universities was a positive experience:

“*OK, I think it was a very good and exciting experience. I think to me as a person – it was the first time I was teaching people across continents using Internet facilities, and so that alone was very exciting for me to see. Because people in Sweden, people from South Africa, and people from Uganda are learning from me. … That was quite exciting. And also, from getting the feedback from some of the students, … they really liked it. Because some of them came on the first day, and after learning that it was on the Internet, they attended from their offices, and they learned everything.*” Teaching staff member

Despite the positive experiences of the real-time sessions, the team realised that the online modality of the interactive lectures or demonstrations covering three sites also made it difficult for students to spontaneously ask questions or give comments. The students had to wait turns to ask questions or use the chat box. Additionally, it was a challenge for the teacher to divide his attention between the students in the face-to-face class and those who joined online. Reading non-verbal cues, recognising potential comments and encouraging speakers were especially difficult as the teacher simultaneously facilitated the discussion and scanned the low-resolution screens showing the online classrooms. These challenges, coupled with occasional interruptions caused by unreliable Internet connections, somewhat limited the dynamics of the discussion across the three sites.

The second part of the course learning activities was the application of new knowledge and skills, building and reinforcing knowledge construction through performing exercises and integrating the lessons into individual assignments. From the teacher’s perspective, “doing” was perceived as a sign of the successful achievement of knowledge acquisition and understanding:

“*You know, whenever we talk about hands-on, practical training, we think of the classroom. But it is quite interesting what people who were not in the classroom and were actually given practical skills were able to do.*” Teaching staff member

Students appreciated the hands-on demonstrations, exercises and individual assignments, which helped them learn new data analysis skills and kept them actively engaged in learning throughout the course. The students reported being motivated to produce high-quality protocols and benefitting from detailed written feedback that they could use to improve their work. However, some students wished that the practical exercises allowed them to use their own real-world problems, thus requesting further customisation of the course:

“*It also would have been useful to insist that each student have a particular diagnostic test they wished to review so that they could have used this as a real example of how to do the review. The course was fine for understanding the basics of diagnostic accuracy reviews, but I think it would have been even more useful if I had been made to apply what I had learned on a daily basis to a real example of my own. The only way one truly learns is to do.*” Evaluation survey respondent

Strong learner support and maximum responsiveness were strategic techniques applied in the course design so that course-related issues could be promptly addressed to prevent frustration among students, especially considering the nature of the course as an experiment. The engagement of the course leader and dedicated course facilitators at each of the remote sites allowed students to receive timely advice and feedback:

“*We had a facilitator we could contact all through the training to enhance our learning.* [It] *was a great strength in terms of helping with understanding and saving time.*” Evaluation survey respondent

The engagement of competent, local teaching facilitators also strengthened personalised feedback through the provision of detailed comments on students’ individual assignments.

However, implementing the course through this approach was not without its challenges. BL requires that course designers develop new competencies and roles to plan a course that combines different teaching and learning modes. The team had only a few people at each site who had to fulfil many different roles and perform multiple responsibilities. At the same time, some key competencies, such as e-learning materials design and BL instruction, were not available. Although the staff were experienced educators, they were new to BL:

“[W]*e are teaching online for the first time, so we are learning as we do.*” Teaching staff member

BL created a larger workload than expected, particularly in adapting content, designing learning activities and coordinating tasks across the three institutions:

“*I think, there is more time involved in this. The reason is* [that] *you are going to teach so many people across continents and people with different backgrounds… and people of different cultures.… It takes a lot of effort to make this course functional. A lot.*” Teaching staff member

### Benefits of the course design and proposed changes

The main benefit of the BL course design according to students was the various forms of flexibility. Synchronous online learning was positively assessed by the students as it offered more flexibility than traditional face-to-face teaching in where and how learning could take place. It enabled participation without disrupting learners’ everyday lives. Some students attending the course at MU, where face-to-face participation was available, valued the option of joining the course online without having to leave their home or office:

“*This allowed me to do the work from my office. I was able to ask questions of the facilitators and get instant answers. This method enabled us to work from our work stations because I would not have been able to obtain sponsorship to travel to either Sweden or South Africa to attend the course. This enabled me to continue with my day-to-day work and attend the workshop as well.*” Evaluation survey respondent

The other form of flexibility – the time when the learning happened – was introduced by the self-directed part of the course (although as we suggest later, the flexibility of self-directed learning could be further increased). Combining the learning activities with these various flexible elements, as well as adding on-site support for learners, gave students at three different universities the ability to fully participate in the course.

The other advantage of this course as cited by students was affordability, as this course did not require spending money on travel or taking time off work and thus losing income-earning opportunities. From the teachers’ point of view, this course implementation offered more efficiency in terms of time and costs which would be required for them to travel to teach this course separately at each of these university sites.

A few changes were proposed for future iterations of the course. The students reported not having “*as much time as* [they] *would have liked to make better use*” of the online discussion forum, readings and additional learning resources. The course’s full-time schedule did not allow much freedom for planning learning outside the classroom, and students had to keep up with the readings for the next session. We propose revising the course duration and spacing the sessions out over time (e.g. once a week) to allow more time on task for asynchronous learning activities and to give students autonomy and flexibility to manage their studying. These changes would make the course better suited to support learner-centred learning.

In line with this comment, overall, the full potential of the Moodle platform was not used due to the short timeline of the course. The platform was initially introduced as a single-entry point for accessing all the materials and communication tools and was intended to provide structure and support as students navigated through the learning materials and assignments. However, use of the platform was limited partially as it was not considered useful for learners’ communication and support between synchronous sessions in this short timeline. Most of the support and instructions was distributed through other means:

“*Most of the information and communication I had already in form of email, so I did not utilise Moodle for that purpose. The content I revisited several times on Moodle, so that was very helpful. One of the assessments was uploaded on Moodle.*” Evaluation survey respondent

We see this limited use of the platform as a missed opportunity to create space for a virtual learning community or a “community of inquiry” across the three universities that could be effective at facilitating further peer-to-peer learning. Meaningful use of the platform and peer-to-peer learning could be promoted by introducing collaborative learning activities utilising the discussion forums or other options for text-based interaction. In addition, replacing some synchronous activities with self-directed activities for learning content via recorded videos integrated with asynchronous discussions on the platform could increase the temporal flexibility of the course. The shorter real-time sessions could then be more efficiently used for in-depth discussions of the content.

## Discussion

The present case study can be described as an implementation of the “awareness/exploration” stage of BL adaptation [50]. During this early stage, the teaching staff members were the main drivers behind adopting BL and were strongly motivated to accommodate the needs of adult learners through flexible solutions [51]. The benefits of BL were recognised at the institutional level, but no institution, with the exception of SU, had formally implemented BL. The participating institutions had relatively good technological infrastructure, including official university e-learning platforms, video-conferencing facilities and available general information technology support. Some BL instructional and technical support was offered within the framework of the ARCADE consortium through technical working groups and experience-sharing workshops, while the faculty had the freedom to experiment with and innovate in their course design.

This early-adopter implementation of BL in HSSR training across three countries was a valuable experience for the entire consortium and its individual partners as, for many, it was their first attempt to engage with BL. Experimenting with this course design allowed the team to further explore the benefits of this approach, while it also helped point out the need for stronger dedicated support and improved Internet connectivity, workload management and opportunities for staff professional development in BL [52]. Recognising these needs, in turn, helped streamline the implementation of BL and continue work on developing courses to build the capacity of staff and students within the ARCADE HSSR consortium.

The institutions played a crucial role in supporting and sustaining this early-adopter effort, but further work is needed to put BL structures in place, such as the adoption of BL strategies, improvement of technical infrastructure and creation of training opportunities and incentives to encourage teaching staff. Some challenges, such as Internet connectivity and the performance of e-learning tools, might lessen in the future as the African regional infrastructure for e-learning is being developed rapidly [53].

Pedagogical design remains a key issue. The course development team had the same challenge as course designers globally [54]: to devise a balanced mix of learning activities and materials that took into account individual learning styles, course objectives, technological solutions, current infrastructure conditions, and teaching staff’s experience [55]. In our case study, the teaching faculty worked from the perspective of social constructivism and were keen to carry over this theoretical approach to BL. Thus, the MADAS was designed as a highly interactive course dependent on synchronous online discussions. Although real-time interaction has been linked to increased students’ course satisfaction in our study and in others [27, 29, 30, 33], learners also valued flexibility. Integrating technology into the course design, such as recorded lectures, has helped introduce greater flexibility and create personalised learning experiences [56]. With increased online content for asynchronous learning, students are free to prioritise their work, proceed at a feasible pace of learning and return to lessons they do not understand, and thus overcome attention and language problems [33]. Increased course duration and time between synchronous sessions could provide more opportunities to engage with the learning materials [27] and allow students more flexibility and independence in their learning. Although these aspects of the course design were not considered while developing the MADAS course, this was a valuable finding that was later implemented in the next courses’ iterations within ARCADE HSSR.

The BL approach can offer flexible access to coursework to a larger number of doctoral students, expanding the impact of training within specialised areas such as MADAS. Few persons in one country might be interested in this specific field, but more can be reached through collaborative, online courses that transcend country borders. Without these efforts, available expertise might remain isolated. In addition, BL can reach students previously unable to participate, including key professionals, women and people with disabilities. Demographic analysis of learners in ARCADE courses showed that a large share of participants in ARCADE BL courses were female (57%), had children younger than 5 years (35%) and combined their studies with employment or research work (60%) [57].

BL also had the added advantage of connecting students from different backgrounds and countries. Despite the occasional problems associated with real-time connections, the students taking part in the MADAS course had a rich experience of educational exchange, similar to participants in other studies [58]. This interaction was important for meaningful collaborative learning [29], while the relationships between students stimulated critical thinking [59] and served as a way to enter a global scientific network [8]. Lengthening the course duration might allow for more ways of connecting the groups, such as discussion forums or social media, support the development of one international community of inquiry [46].

The course design allowed students to receive personalised feedback and detailed written comments on their study protocols from the main lecturers and on-site tutors, which students found useful for their learning [37]. This feedback also served as cross-institutional mentoring [60], a key to students’ progress in their careers [61].

Our case study has highlighted the successes and challenges of implementing a BL course, reporting on a sample of 18 students’ survey responses and qualitative data from course participant observation and feedback from teaching staff and students. A larger sample and more extensive quantitative data could permit a more detailed analysis of students’ experience. However, this sample was sufficient for this paper’s focus on analysing the entire course experience from the perspectives of administrators, lecturers and students. This study was also limited by using project staff as evaluators, which could influence the data interpretation. We attempted to counter this possibility by including staff more peripherally involved in the implementation and preparation of the paper.

## Conclusions

The MADAS course described in this paper is one of only a few examples of southern expertise delivered via BL to northern and southern partners. Transforming a face-to-face course into a blended course and retaining the full potential of the original course requires concerted effort and considerable time, as well as dedicated BL technological and pedagogical support. Despite the initial challenges experienced during the course, the experiment showed the promise of BL for capacity building in HSSR in SSA. BL can facilitate access to high-level research training and offer an engaging format and personalised learning experience. This approach enables involving the best learning expertise in the field alongside learning from peers across the globe.

## Box 1. Overview of MADAS content and learning activities

Module 1

Overview of systematic reviews and meta-analyses. Introduction to diagnostic tests and diagnostic accuracy studies (DAS).

Module 2

Meta-analysis study protocol: defining the problem and test of interest. Introduction, objectives/research questions, methods. Defining the article selection criteria.

*Homework*: Selection of one test for meta-analysis and definition of the selection criteria for the identification of eligible articles.

Module 3

Use of online databases to search for articles. QUADAS and other tools for assessing the quality of articles and final article selection.

*Skills practice session*: Literature search for articles on the selected diagnostic test.

*Homework*: Reading, evaluation and selection of articles to be included in the meta-analysis.

Module 4

Selection and prioritisation of study variables. Statistical data analysis for pooled sensitivity, specificity and results interpretation. Analysis for heterogeneity among the primary articles and reports included in the meta-analysis.

*Skills practice session*: Data extraction and summarisation.

*Homework*: Data analysis and submission of the results.

Module 5

Development of a meta-analysis study manuscript. Review of STARD and other tools for reporting DAS. Biases and challenges in conducting and publishing a meta-analysis study.

Examination

Development of a draft meta-analysis protocol of one test method (40% of the mark) and final test with multiple-choice questions (60% of the mark).

## Box 2. Seven principles of effective teaching

1. Student–faculty contact – frequent student–faculty contact inside and outside class

2. Cooperation among students – promotion of collaborative and social learning

3. Active learning – use of techniques to promote active student involvement in the construction and acquisition of knowledge

4. Prompt feedback – appropriate, timely feedback on student performance

5. Time on task – “Time plus energy equals learning”; realistic allocation of time for effective teaching and learning activities

6. High expectations – “Expect more, and you will get more”; setting high expectations for student performance

7. Respect for diverse talents and ways of learning – including a variety of learning activities to accommodate different learning styles

Adapted from Chickering and Gamson [47] and Bangert [48]

## Abbreviations

ARCADE HSSR: African regional capacity development for health systems and services research
BL: blended learning
HSSR: health systems and services research
KI: Karolinska Institutet
LMIC: low- and middle-income countries
MADAS: meta-analysis of diagnostic accuracy studies
MU: Makerere University
SSA: sub-Saharan Africa
SU: Stellenbosch University
